# Sexual violence across gender identities and sexual orientations: a stratified, population-based, cross-sectional study among young people aged 16–29 years in Sweden

**DOI:** 10.1186/s12889-025-22970-3

**Published:** 2025-05-22

**Authors:** Matilda Löfström-Bredell, Eva Åkerman, Renita Sörensdotter, Sara Ström, Henrik Källberg, Marie Klingberg-Allvin

**Affiliations:** 1 Department of Women’s and Children’s Health, Karolinska Institutet, Stockholm, Sweden; 2 Centre for Gender Research, National Centre for Knowledge on Men’s Violence against Women, Uppsala University, Uppsala, Sweden; 3 Public Health Agency of Sweden (Folkhälsomyndigheten), Solna, Sweden

**Keywords:** Sexual violence, Forced penetration, Sexual assault, Online sexual abuse, Image-based sexual abuse, Young people, Gender identity, Sexual orientation, LGBTQ + individuals, Sexual and reproductive health and rights (SRHR)

## Abstract

**Background:**

Sexual violence is a critical and preventable global threat to public health, sexual and reproductive health and rights, and gender equality. Studies have shown that sexual violence among young people in Sweden is prevalent, with lesbian, gay, and bisexual individuals facing greater exposure than their heterosexual peers do. Research has also indicated that nonbinary youth report high levels of sexual violence. However, population-based studies in Sweden on sexual violence across gender identities and sexual orientations among those aged 16 to 29 years remain limited.

**Aim:**

This study aims to investigate the lifetime prevalence of being penetrated without consent, of being physically assaulted during sex, and of exposure to online sexual abuse and image-based sexual abuse, as well as their associations with gender identities and sexual orientations among young people in Sweden. This study also aims to examine the categories of perpetrators of sexual violence reported by young people.

**Methods:**

A population-based, cross-sectional survey using stratified random sampling (response rate = 23.7%) yielded a sample of 9,430 respondents. The survey data were complemented with national register data from Statistics Sweden. Statistical analyses included chi-square tests and logistic regression analyses.

**Results:**

Young women and nonbinary individuals reported higher levels of all four types of violence compared to young men, with the categories of perpetrators differing by type of violence. Compared to heterosexual men, bisexual and heterosexual women were more likely to experience the four types of sexual violence. Additionally, lesbian women, along with gay and bisexual men, faced higher odds of exposure to three types of violence compared to heterosexual men. Our results indicate that, compared to nearly all groups, bisexual women are particularly vulnerable, showing the highest prevalence of all forms of sexual violence.

**Conclusions:**

In summary, our study reveals inequities in lifetime exposure to four types of sexual violence across gender identities and sexual orientations, revealing insights into the vulnerability of bisexual, lesbian, and heterosexual women, gay and bisexual men, as well as nonbinary individuals. To eliminate sexual violence in Sweden, measures involving stakeholders at all levels are needed, focusing on tailored prevention efforts and inclusive policies that address the vulnerabilities of these groups while promoting equity for all young people.

**Supplementary Information:**

The online version contains supplementary material available at https://doi.org/10.1186/s12889-025-22970-3.

## Background

Sexual violence is a significant and preventable global threat to public health and young people’s sexual and reproductive health and rights (SRHR), causing enduring and complex impacts [1–4]. The World Health Organization defines sexual violence as “any sexual act, attempt to obtain a sexual act, or other act directed against a person’s sexuality using coercion, by any person regardless of their relationship to the victim, in any setting.” [5]. The definition includes both physical and digital forms of sexual violence, such as physical assault or coercing someone into engaging in sexual acts in front of a web camera.

All forms of sexual violence violate bodily integrity and autonomy and undermine the fulfilment of sexual and reproductive rights. According to the Guttmacher-Lancet Commission’s definition of SRHR, realising rights is vital for achieving good sexual and reproductive health [6]. Furthermore, every individual has the right to safe and pleasurable sexual experiences free from violence, discrimination and coercion [6]. Physical assault during sex, such as being slapped, hit, or held in a chokehold against one’s will, constitutes a violation of that right. Despite increased media attention in recent years [7, 8], physical assault during sex has been underrepresented in population-based studies.

As vulnerability to sexual violence is not evenly distributed [9, 10], some populations face greater risks due to intersecting factors, such as age, gender identity, sexual orientation, and socioeconomic status [11]. Global estimations reveal that about a third of women and girls have a lifetime experience of sexual violence, highlighting the widespread prevalence and gender differences [12–14]. According to international indices on gender equality, Sweden and other Nordic countries are among the most gender-equal nations globally while simultaneously showing excessively high levels of violence against women, referred to as the ‘Nordic paradox’ [15]. The theory is based on the premise that gender equality cannot be fully reached while violence against women and girls remains high [2]. In Sweden, school-based sexuality education has been in place since 1955 [16], with a particular emphasis on sexual consent introduced in 2022 [17], alongside a 2016 national policy aimed at preventing violence [18] and modifications in the Swedish Code of Statutes, founded on the principle that consent is the cornerstone of sex [19]. Nevertheless, studies indicate that various forms of sexual violence are prevalent among young people, particularly affecting girls more than boys [20–24]. Additionally, reports from Swedish government agencies have indicated that nonbinary youth [25] and young lesbian, gay and bisexual (LGB) [26, 27] exhibit heightened vulnerability to sexual violence. This finding is further supported by international studies [28–31], which demonstrate that LGB people, along with those who have experienced discrimination based on their sexual orientation, face a greater risk of encountering sexual violence [32–34].

Statistically, it is common for people who have been subjected to sexual violence to know the perpetrator since the assault often occurs within relationships by a current or former partner, known as intimate partner violence or dating violence [12, 35]. Nonetheless, perpetrators of sexual violence are not limited to partners, and some studies have shown that, for instance, friends and acquaintances are equally common groups of perpetrators [27]. Other perpetrators of sexual violence include family members, relatives, and strangers [12].

International initiatives have long emphasised the urgent need to eliminate sexual violence, recognising it as a public health crisis and a barrier to gender equality, health equity, and sexual and reproductive health and rights [1–4, 36, 37]. Despite these efforts, high prevalence rates persist globally and nationally, cutting across socioeconomic, cultural and geographical boundaries [12, 13]. This highlights the importance of identifying vulnerable groups to develop tailored prevention strategies. However, there is a lack of population-based research examining the complex relationships between lifetime prevalence of sexual violence, gender identities, and sexual orientations among young people. This study addresses this gap by analysing the factors contributing to this essential body of knowledge.

## Aim

This study aims to investigate the prevalence of being penetrated without consent, of being physically assaulted during sex, exposure to online sexual abuse and image-based sexual abuse among young people in Sweden and to examine the associations between these forms of sexual violence and sociodemographic factors, such as gender identities and sexual orientations. This study also aims to investigate the categories of perpetrators of sexual violence reported by young people.

## Methods

### Study design, survey and sample

The UngKAB23 study was conducted by the Public Health Agency of Sweden and aimed to explore young people’s sexual and reproductive health and rights. The survey included 96 items on sexual violence, safer sex practices, sexuality education, etc., of which 13 were selected for this study (Appendix 1). Statistics Sweden reviewed the survey to minimise measurement errors (e.g., assessing items and instructions) and conducted seven cognitive interviews with young people, further informing its development. The Swedish Total Population Register provided a natural sampling frame, from which a stratified random sample of 39,894 individuals was drawn across 42 strata defined by legal gender, age group, and geographical region in Sweden. The inclusion criteria specified that participants must be between 16 and 29 years old, hold a Swedish personal identification number, and comprehend Swedish, as the survey was administered in that language.

### Data collection

Statistics Sweden (SCB) collected data on behalf of the Public Health Agency of Sweden from October 2023 to January 2024. The survey was only available on digital platforms. Invitations were sent via digital or conventional mail, with five total mailings, the third and fifth by conventional mail only. All included a survey login link. Digital mailbox users needed e-identification, while conventional mail recipients could also use a username and password. Of 9,444 respondents (23.7% response rate), 9430 were included, excluding 14 due to partial dropout related to gender identity.

### Measures

The outcome variables included four types of lifetime prevalence of sexual violence: being penetrated without consent, being physically assaulted during sex, exposure to online sexual abuse, and image-based sexual abuse. The participants who had experienced violence were asked a follow-up question regarding perpetrators, which included multiple response options (Appendix 1).

The exposure variables included gender identity and sexual orientation. We also developed a new variable with six categories: “Heterosexual women”, “Heterosexual men”, “Lesbian women”, “Gay men”, “Bisexual women”, and “Bisexual men”. Furthermore, the background and control variables included three survey variables: sexual debut, relationship status and sexual orientation-related discrimination (Appendix 1). Additionally, we included age, origin, and disposable household income from SCB’s Registry (Appendix 2).

### Statistical analyses

We used descriptive statistics with unweighted data to summarise sample characteristics stratified by gender identity and sexual orientation (Table 1). Being penetrated without consent, online sexual abuse, and image-based sexual abuse were presented to all participants (*n* = 9,430), whereas physical assault during sex was presented only to those with sexual experience (*n* = 7,000). Chi-square tests (p-value < 0.05) were used to examine the prevalence of sexual violence (Tables 2 and 3) and reported perpetrator categories (Table 4), including all response options (“Yes”, “No”, and “Not sure”). The nonresponse rate of the items (missing data) ranged from 0.1 to 0.7%, with the highest value being for sexual orientation, and these were excluded from all analyses. We dichotomised the outcome variables for all logistic regression analyses into “Yes” and “No”. We excluded “Not sure” responses, resulting in a total exclusion rate of 3.6%, of which “Not sure” accounted for 3.4% (being penetrated without consent). The corresponding exclusion rates for physical assault during sex totaled 3.1%, with “Not sure” making up 3.0%; for online sexual abuse, the total exclusion rate was 3.2%, with “Not sure” comprising 3.1%; and image-based sexual abuse, the total exclusion rate was 3.1%, where “Not sure” made up 3.0%. Crude logistic regression models were used to assess the associations (odds ratios) between each type of sexual violence and gender identity (reference category: young men) (Table 5). Adjusted logistic regression models included age group, origin, disposable household income, sexual orientation, and sexual orientation-related discrimination (Table 5).

Crude logistic regression models were used to assess the associations between gender/sexual orientation (reference category: heterosexual men) and each type of sexual violence. The second model was adjusted for age group, origin, disposable household income and sexual orientation-related discrimination (Table 3). We present 95% confidence intervals (CIs) and consider a p-value of less than 0.05 to be statistically significant. All analyses were conducted in Stata 16.1 (StataCorp, College Station, Texas, USA).

### Ethical approval

The Swedish Ethical Review Authority provided ethical approval on August 28th, 2023 (ref. no. 2023-04158-01).

## Results

### Sociodemographic characteristics

Among the respondents (*n* = 9,430), 55.7% identified as women, 41.7% as men, and 2% as nonbinary or did not want to categorise themselves. In total, 22.6% had another sexual orientation than heterosexual; for example, 9.2% were bisexual, and 2.7% were lesbian or gay. Most participants were born in Sweden to Swedish-born parents (71.6%) and had sexually debuted (74.3%). A total of 4.5% had experienced sexual orientation-related discrimination in the previous six months (Table 1).

**Table 1:** Sociodemographic characteristics of respondents (*n* = 9,430)

Background variables	Young women *N* = 5,253 (55.7%) ^a^			Young men *n* = 3,935 (41.7%) ^a^			Young nonbinary+ *n* = 192 (2.0%) ^a^			Total ^a^ *n* = 9,430
	Heterosexual 3,905 (84) ^b^	Lesbian 113 (2) ^b^	Bisexual 644 (14) ^b^	Heterosexual 3,340 (92) ^b^	Gay 111 (3) ^b^	Bisexual 173 (5) ^b^	Heterosexual 16 (8.3) ^b^	Homosexual 23 (12.0) ^b^	Bisexual 36 (18.8) ^b^	All groups
Age group										
*16–19*	1,230 (31.5)	30 (26.5)	173 (26.9)	1,004 (30.1)	18 (16.2)	42 (24.3)	5 (31.3)	13 (56.5)	12 (33.3)	2931 (31.1)
*20–24*	1,248 (32.0)	50 (44.3)	250 (38.8)	1,108 (33.2)	45 (40.5)	71 (41.0)	6 (37.5)	7 (30.4)	17 (47.2)	3178 (33.7)
*25–29*	1,427 (36.5)	33 (29.2)	221 (34.3)	1,228 (36.8)	48 (43.2)	60 (34.7)	5 (31.3)	3 (13.0)	7 (19.4)	3321 (35.2)
Origin										
*Swedish-born with*:										
*Swedish-born parents*	2,856 (73.4)	79 (69.9)	482 (75.1)	2,422 (72.8)	65 (59.1)	123 (71.1)	10 (62.5)	15 (65.2)	3 (8.3)	6726 (71.6)
*One foreign-born parent*	348 (8.9)	15 (13.3)	74 (11.5)	305 (9.2)	15 (13.6)	23 (13.3)	^c^	^c^	^c^	882 (9.4)
*Two foreign-born parents*	234 (6.0)	7 (6.2)	26 (4.0)	198 (5.9)	7 (6.4)	5 (2.9)	^c^	^c^	3 (8.3)	525 (5.6)
*Foreign-born*	453 (11.6)	12 (10.6)	60 (9.3)	403 (12.1)	23 (20.9)	22 (12.7)	6 (37.5)	5 (21.7)	28 (77.8)	1256 (13.4)
In a relationship										
*Yes*	2,196 (57.6)	44 (38.9)	358 (57.2)	1,469 (44.8)	36 (33.3)	70 (41.4)	9 (64.3)	7 (30.4)	23 (69.7)	4550 (49.4)
*No*	1,617 (42.4)	69 (61.1)	268 (42.8)	1,813 (55.2)	72 (66.7)	99 (58.6)	5 (35.7)	16 (69.6)	10 (30.3)	4666 (50.6)
Had their sexual debut										
*Yes*	3,173 (81.4)	74 (65.5)	547 (84.9)	2,391 (71.7)	84 (76.4)	131 (75.7)	12 (75.0)	12 (52.2)	25 (69.4)	6993 (74.3)
*No*	723 (18.6)	39 (34.5)	97 (15.1)	943 (28.3)	26 (23.6)	42 (24.3)	4 (25.0)	11 (47.8)	11 (30.6)	2417 (25.7)
Experienced sexual orientation-related discrimination during the last 6 months										
*Yes*	10 (0.3)	51 (45.1)	98 (15.2)	43 (1.29)	37 (33.6)	26 (15.0)	3 (18.8)	13 (56.5)	17 (47.2)	424 (4.5)
*No*	3,891 (99.7)	62 (54.9)	545 (84.8)	3,292 (98.7)	73 (66.4)	147 (85.0)	13 (81.3)	10 (43.5)	19 (52.8)	8985 (95.5)
Disposable household income										
*Lowest (10th percentile)*	756 (19.4)	23 (20.4)	127 (19.7)	713 (21.4)	25 (22.5)	35 (20.2)	^c^	8 (34.8)	7 (19.4)	1888 (20.0)
*Low (25th percentile)*	766 (19.6)	20 (17.7)	150 (23.3)	637 (19.1)	25 (22.5)	39 (22.5)	6 (37.5)	^c^	8 (22.2)	1886 (20.0)
*Middle (50th percentile)*	755 (19.3)	26 (23.0)	142 (22.1)	658 (19.7)	20 (18.0)	39 (22.5)	4 (25.0)	^c^	8 (22.2)	1886 (20.0)
*High (75th percentile)*	848 (21.7)	16 (14.2)	116 (18.0)	636 (19.4)	19 (17.1)	27 (15.6)	4 (25.0)	4 (17.4)	^c^	1886 (20.0)
*Highest (90th percentile)*	780 (20.0)	28 (24.8)	109 (16.9)	696 (20.8)	11 (19.8)	33 (19.1)	^c^	7 (30.4)	11 (30.6)	1884 (20.0
Forced penetration										
*Yes*	829 (21.3)	20 (17.7)	233 (36.2)	39 (1.2)	11 (9.9)	12 (6.9)	1 (18.8)	5 (21.7)	7 (19.4)	1302 (13.8)
*No*	2877 (73.8)	90 (79.7)	369 (57.4	3276 (98.3)	95 (85.6)	160 (92.5)	11 (68.8)	17 (73.9)	27 (75.0)	7790 (82.8)
*Not sure*	193 (5.0)	^c^	41 (6.4)	17 (0.5)	5 (4.5)	^c^	^c^	^c^	^c^	321 (3.4)
Physical assault during sex										
*Yes*	451 (14.2)	13 (17.6)	158 (28.9)	53 (2.2)	9 (10.6)	8 (6.11)	^c^	4 (33.3)	6 (24.0)	802 (11.4)
*No*	2606 (82.0)	58 (78.4)	359 (65.8)	2326 (97.0)	74 (87.1)	120 (91.6)	12	7 (58.3)	15 (60.0)	5998 (85.6)
*Not sure*	123 (3.9)	^c^	29 (5.3)	18 (0.8)	^c^	3 (2.3)	^c^	^c^	4 (16.0)	210 (3.0)
Online sexual abuse										
*Yes*	456 (11.7)	16 (14.2)	133 (20.7)	65 (2.0)	13 (11.7)	16 (9.3)	^c^	4 (17.4)	5 (13.9)	838 (8.9)
*No*	3262 (83.7)	92 (81.4)	485 (75.3)	3246 (97.3)	95 (85.6)	149 (86.1)	15 (93.8)	18 (78.3)	29 (80.6)	8292 (88.1)
*Not sure*	180 (4.6)	5 (4.4)	26 (4.0)	25 (0.8)	3 (2.7)	8 (4.6)	^c^	^c^	^c^	287 (3.1)
Non-consensual sharing of sexual content										
*Yes*	398 (10.2)	12 (10.6)	96 (14.9)	131 (3.9)	8 (7.2)	11 (6.4)	^c^	^c^	5 (13.9)	733 (7.8)
*No*	3365 (86.3)	97 (85.8)	521 (81.0)	3157 (94.7)	94 (84.7)	157 (90.8)	14 (87.5)	20 (87.0)	26 (72.2)	8404 (89.2)
*Not sure*	137 (3.5)	4 (3.5)	26 (4.0)	47 (1.4)	9 (8.1)	5 (2.9)	^c^	^c^	5 (13.9)	280 (3.0)

Note. ^a^ Descriptive statistics are calculated using unweighted data based on all gender identities, including those not listed above: young people who do not know their gender identity (*n* = 50, 0.5%)

^b^ Descriptive statistics are calculated using unweighted data based on all sexual orientations, including those not listed above: pansexuals (*n* = 160, 1.7%), asexuals (*n* = 109, 1.2%), those who do not know (*n* = 347, 3.7%), and those who do not want to categorise themselves (*n* = 389, 4.2%)

^c^ The number of respondents was too few to report (less than three)

### Prevalence of four types of sexual violence

We found that young women were 11 times more likely than young men to report a lifetime prevalence of being penetrated without consent. Compared with young men, young women also had a greater prevalence of the three types of sexual violence. Furthermore, nonbinary+ youth had a greater prevalence of being penetrated without consent, physically assaulted during sex and exposed to online sexual abuse than young men did. Bisexuals and pansexuals reported the highest prevalence of being penetrated without consent among the sexual orientation groups (Table 2).

**Table 2:** Lifetime prevalence of four types of sexual violence among young people

Characteristics	Lifetime exposure to:	Frequency, ‘yes’-response	Prevalence rate % (95% CI) ^a^
**Gender identity (n = 9**,**430)**			
*Young women* (*n* = 5,246)	Being penetrated without consent	1,193	22.7 (21.6–23.9)
	Being physically assaulted during sex	696	16.8 (15.7–18.0)
	Exposure to online sexual abuse	690	13.2 (12.3–14.1)
	Exposure to image-based sexual abuse	559	10.7 (9.8–11.5)
*Young men* (*n* = 3,925)	Being penetrated without consent	73	1.9 (1.5–2.3)
	Being physically assaulted during sex	79	2.9 (2.3–3.6)
	Exposure to online sexual abuse	112	2.8 (2.4–3.4)
	Exposure to image-based sexual abuse	160	4.1 (3.5–4.7)
*Young nonbinary+* (*n* = 192)	Being penetrated without consent	31	16.1 (11.6–22.0)
	Being physically assaulted during sex	24	22.9 (15.8-–31.8)
	Exposure to online sexual abuse	32	16.7 (12.0–22.6)
	Exposure to image-based sexual abuse	13	6.8 (4.0–11.3)
*Young people who do not know their gender identity* (*n* = 50)	Being penetrated without consent	5	10.0 (4.2–21.9)
	Being physically assaulted during sex	3	15.8 (5.2–39.2)
	Exposure to online sexual abuse	4	8.0 (3.0–19.5)
	Exposure to image-based sexual abuse	^b^	^b^
**Sexual orientation (n = 9**,**381)**			
*Young heterosexuals* (*n* = 7,264)	Being penetrated without consent	871	12.0 (11.3–12.8)
	Being physically assaulted during sex	504	9.0 (8.3–9.8)
	Exposure to online sexual abuse	522	7.2 (6.6–7.8)
	Exposure to image-based sexual abuse	531	7.3 (6.7–7.9)
*Young lesbians/gays* (*n* = 249)	Being penetrated without consent	36	14.5 (10.6–19.4)
	Being physically assaulted during sex	26	15.1 (10.5–21.3)
	Exposure to online sexual abuse	33	13.3 (9.6–18.1)
	Exposure to image-based sexual abuse	22	8.8 (5.9–13.1)
*Young bisexuals* (*n* = 863)	Being penetrated without consent	254	29.5 (26.5–32.6)
	Being physically assaulted during sex	172	24.3 (21.3–27.6)
	Exposure to online sexual abuse	156	18.1 (15.6–20.8)
	Exposure to image-based sexual abuse	112	13.0 (10.9–15.4)
*Young pansexuals* (*n* = 160)	Being penetrated without consent	48	30.0 (23.4–37.5)
	Being physically assaulted during sex	36	28.8 (21.5–37.3)
	Exposure to online sexual abuse	42	26.3 (20.0–33.6)
	Exposure to image-based sexual abuse	21	13.1 (8.7–19.3)
*Young asexuals* (*n* = 109)	Being penetrated without consent	11	10.1 (5.7–17.3)
	Being physically assaulted during sex	7	21.9 (10.8–21.1)
	Exposure to online sexual abuse	9	8.3 (4.4–15.1)
	Exposure to image-based sexual abuse	^b^	^b^
*Young people who do not know their sexual orientation* (*n* = 347)	Being penetrated without consent	41	11.8 (8.8–15.7)
	Being physically assaulted during sex	29	15.3 (10.8–21.1)
	Exposure to online sexual abuse	39	11.2 (8.3–15.0)
	Exposure to image-based sexual abuse	20	5.8 (3.8–8.8)
*Young people who do not want to categorise themselves (sexually)* (*n* = 389)	Being penetrated without consent	39	10.0 (7.4–13.4)
	Being physically assaulted during sex	25	14.5 (10.0–21.1)
	Exposure to online sexual abuse	36	9.3 (6.8–12.6)
	Exposure to image-based sexual abuse	25	6.4 (4.4–9.3)

Note. Chi-square analysis was performed with unweighted data and 95% confidence intervals (CIs)

^a^ The proportions were calculated based on all of the response options: “Yes,” “No,” and “Not sure.” Only the “Yes” responses are presented in the table. ^b^ The number of respondents was too small to report (fewer than three)

Compared with all groups, bisexual women reported the highest prevalence of being penetrated without consent, as well as the highest prevalence of physical assault during sex, online sexual abuse, and image-based sexual abuse, except for lesbian women. We also observed that more than every sixth heterosexual and lesbian woman had been penetrated without consent and physically assaulted during sex, and more than every tenth lesbian and heterosexual woman had been exposed to online sexual abuse and image-based sexual abuse. Similarly, more than every tenth gay man had been penetrated without consent and physically assaulted during sex, and every tenth gay and bisexual man had experienced online sexual abuse. The heterosexual men reported the lowest levels of the four of sexual violence (Table 3).

**Table 3:** Associations between sexual violence, gender identity and sexual orientation

	Exposure variables	The proportion exposed to the four types of sexual violence % (CI)	Crude model OR (95% CI)	Adjusted model AOR (95% CI) ^a^
Being penetrated without consent				
*Gender identity and sexual orientation*	Man & heterosexual	1.2 (0.9–1.6)	1 (ref.)	1 (ref.)
	Man & gay	10.4 (5.8–17.8)	9.7 (4.8–19.6)^**^	8.0 (3.9–16.5)^**^
	Man & bisexual	7.0 (4.0–11.9)	6.3 (3.2–12.3)^**^	5.4 (2.8–10.7)^**^
	Woman & heterosexual	22.4 (21.1–23.7)	24.2 (17.5–33.5)^**^	25.6 (18.5–35.5)^**^
	Woman & lesbian	18.2 (12.0–26.5)	18.7 (10.5–33.3)^**^	14.7 (8.0–27.1)^**^
	Woman & bisexual	38.7 (34.9–42.7)	53.0 (37.2–75.5)^**^	49.4 (34.4–70.9)^**^
Physical assault during sex				
*Gender identity and sexual orientation*	Man & heterosexual	2.2 (1.7–2.9)	1 (ref.)	1 (ref.)
	Man & gay	10.8 (5.7–19.5)	5.3 (2.5–11.2)^**^	3.9 (1.8–8.5)^**^
	Man & bisexual	6.3 (3.2–12.0)	2.9 (1.4–6.3)^***^	2.4 (1.1–5.3)^***^
	Woman & heterosexual	14.8 (13.5–16.1)	7.6 (5.7–10.2)^**^	7.8 (5.8–10.4)^**^
	Woman & lesbian	18.3 (10.9–29.0)	9.8 (5.1–19.0)^**^	6.5 (3.2–13.1)^**^
	Woman & bisexual	30.6 (26.7–34.7)	19.3 (13.9–26.9)^**^	17.1 (12.2–24.1)^**^
Online sexual abuse				
*Gender identity and sexual orientation*	Man & heterosexual	2.0 (1.5–2.5)	1 (ref.)	1 (ref.)
	Man & gay	12.0 (7.1–19.6)	6.8 (3.6–12.8)^**^	4.9 (2.5–9.4)^**^
	Man & bisexual	9.7 (6.9–15.2)	5.4 (3.0–9.5)^**^	4.5 (2.5–8.0)^**^
	Woman & heterosexual	12.3 (11.2–13.4)	7.0 (5.4–9.1)^**^	7.0 (5.4–9.2)^**^
	Woman & lesbian	14.8 (9.3–22.8)	8.7 (4.8–14.6)^**^	5.2 (2.8–9.7)^**^
	Woman & bisexual	21.5 (18.5–24.9)	13.7 (10.0–18.7)^**^	11.3 (8.2–15.6)^**^
Image-based sexual abuse				
*Gender identity and sexual orientation*	Man & heterosexual	4.0 (3.4–4.7)	1 (ref.)	1 (ref.)
	Man & gay	7.8 (4.0–14.9)	2.1 (1.0–4.3)	1.4 (0.6–3.1)
	Man & bisexual	6.5 (3.7–11.4)	1.7 (0.9–3.2)	1.3 (0.7–2.6)
	Woman & heterosexual	10.6 (9.6–11.6)	2.9 (2.3–3.5)^**^	2.9 (2.3–3.5)^**^
	Woman & lesbian	11.0 (6.4–18.4)	3.0 (1.6–5.7)^***^	1.7 (0.9–3.3)
	Woman & bisexual	15.6 (12.9–18.6)	4.4 (3.4–5.9)^**^	3.6 (2.6–4.8)^**^

Note. Logistic regression analysis presented with unweighted odds ratios (ORs) and adjusted odds ratios (AORs) in percentages with 95% confidence intervals (CIs). ^a^ Adjusted for age group, origin, household disposable income, and sexual orientation-related discrimination. ^**^Statistically significant at *p* < 0.001. ^***^Statistically significant at *p* < 0.05

### Perpetrators of sexual violence

Most young women and men who had been penetrated without consent identified the perpetrator as an acquaintance or partner. In cases of physical assault during sex, the most commonly reported perpetrator was a temporary sex partner. For online sexual abuse, the majority reported the perpetrator as someone unknown. In cases of image-based sexual abuse, a greater proportion of young women reported either a partner or an acquaintance as the perpetrator than young men. In contrast, more young men reported perpetration by someone unknown. A majority of nonbinary + youth reported partners as perpetrators of penetration without consent, temporary sex partners as perpetrators of physical abuse during sex, and someone unknown as perpetrators of online abuse and image-based sexual abuse (Table 4).

**Table 4:** Proportions of reported perpetrators of four types of sexual violence

Type of sexual violence	Categories of perpetrators	Young women % (CI)	Young men % (CI)	Young nonbinary+ % (CI)
Being penetrated without consent	A current or former partner	42.8 (40.0–45.7)	31.5 (21.7–43.3)	54.8 (36.5–72.0)
	A family member	2.9 (2.1–4.1)	11.0 (5.6–20.4) ^*^	^a^
	Someone close (other than above)	4.8 (3.7–6.1)	8.2 (3.7–17.4)	12.9 (4.6–31.1)
	An acquaintance	44.9 (42.1–47.8)	38.4 (27.7–50.2)	38.7 (22.7–57.6)
	Someone unknown	23.4 (21.1–26.0)	26.0 (17.1–37.5)	12.9 (4.6–31.1)
Physical assault during sex	A current or former partner	43.1 (39.5–46.8)	36.7 (26.7–48.1)	45.8 (26.3–66.8)
	A temporary sex partner	59.6 (55.9–63.2)	55.7 (44.6–66.2)	50.0 (29.7–70.3)
	An acquaintance	20.5 (17.7–23.7)	22.8 (14.7–33.6)	29.2 (13.7–51.5)
Online sexual abuse	A current or former partner	28.3 (25.0–31.7)	19.6 (13.2–28.2)	18.8 (8.3–37.1)
	A family member	1.0 (0.5–2.1)	^a^	^a^
	Someone close (other than above)	3.6 (2.5–5.3)	^a^	^a^
	An acquaintance	33.3 (29.9–36.9) ^*^	23.2 (16.3–31.9)	25.0 (12.5–43.7)
	Someone unknown	56.5 (52.8–60.2)	66.1 (56.7–74.3)	75.0 (56.3–87.5)
Image-based sexual abuse	A current or former partner	27.5 (24.0–31.4) ^*^	11.3 (7.2–17.2)	^a^
	A family member	0.9 (0.4–2.1)	^a^	^a^
	Someone close (other than above)	5.9 (4.2–8.2)	5.6 (2.9–10.5)	^a^
	An acquaintance	44.4 (40.3–48.5) ^*^	31.9 (25.1–39.6)	46.2 (19.5–75.2)
	Someone unknown	36.7 (32.8–40.8)	65.6 (57.9–72.6) ^*^	76.9 (42.8–93.7)

Note. Descriptive statistics are presented as unweighted percentages (%) and 95% confidence intervals (CIs). Chi2-tests with a p-value of < 0.05 were conducted on differences in reported perpetrators between women and men (nonbinary + individuals were excluded due to low frequencies). ^a^ The number of respondents was too small to report (fewer than three). ^*^ Statistically significant at *p* < 0.05

### Associations between sexual violence and gender identity

The logistic regression analysis revealed that young women had 18 times higher odds of being penetrated without consent than young men did (adjusted odds ratio [AOR] 18.2, 95% confidence interval [CI]: 14.0–23.6, p-value < 0.001). Additionally, nonbinary + youth faced higher odds of being ten times more likely to be penetrated without consent than young men did (AOR 10.7, 95% CI: 5.5–20.7, p-value < 0.001). Furthermore, young women and nonbinary + youth were substantially more likely to experience physical assault during sex and online sexual abuse than their male counterparts were (Table 5).

**Table 5:** Associations between gender identity and sexual violence

Outcome variables: Types of sexual violence	Exposure variable: Gender identity	Crude model OR (95% CI)	Adjusted model AOR (95% CI) ^a^
Being penetrated without consent	Young men	1 (ref.)	1 (ref.)
	Young women	16.5 (13.0–21.0)^**^	18.2 (14.0–23.6)^**^
	Young nonbinary+	10.9 (6.9–17.1)^**^	10.7 (5.5–20.7)^**^
Physical assault during sex	Young men	1 (ref.)	1 (ref.)
	Young women	7.1 (CI: 5.6–9.0)^**^	7.0 (5.4–9.0)^**^
	Young nonbinary+	11.3 (6.7–18.9)^**^	5.0 (2.3–11.0)^**^
Online sexual abuse	Young men	1 (ref.)	1 (ref.)
	Young women	5.4 (CI: 4.4–6.6)^**^	5.4 (4.3–6.8)^**^
	Young nonbinary+	7.1 (4.6–10.9)^**^	2.7 (1.3–5.6)^**^
Image-based sexual abuse	Young men	1 (ref.)	1 (ref.)
	Young women	2.9 (CI: 2.4–3.4)^**^	2.9 (2.3–3.3)^**^
	Young nonbinary+	1.9 (1.0–3.4)^***^	1.8 (0.8–3.9)

Note. Logistic regression analysis is presented with unweighted odds ratios (ORs) and adjusted odds ratios (AORs) in percentages with 95% confidence intervals (CIs). ^a^ Adjusted for sexual orientation, age group, origin, household disposable income, and sexual orientation-related discrimination. ^**^Statistically significant at *p* < 0.001. ^***^Statistically significant at *p* < 0.05

### Associations between sexual violence, gender identity and sexual orientation

Compared with heterosexual men, the odds of being penetrated without consent, physical assault during sex and online sexual abuse were significantly higher among bisexual, heterosexual and lesbian women, as well as gay and bisexual men. Furthermore, bisexual women had significantly higher odds of being penetrated without consent than did all other groups, except for heterosexual women, and significantly higher odds of experiencing physical assault during sex than did all other groups except for lesbian women. Additionally, bisexual and heterosexual women were more likely to be exposed to image-based sexual abuse than heterosexual men were. There were no significant differences in the odds of experiencing the four types of sexual violence between bisexual and gay men (Table 3).

## Discussion

We aimed to examine the prevalence of four types of sexual violence and their associations with gender identities and sexual orientations, as well as to investigate categories of perpetrators. Our analysis revealed that young women and nonbinary + individuals reported higher levels of the four types of violence than young men did, with perpetrator categories varying by type of violence. Compared with heterosexual men, bisexual and heterosexual women were more likely to be exposed to the four types of sexual violence. Conversely, lesbian women, along with gay and bisexual men, faced increased odds of exposure to three types of violence in comparison to heterosexual men. Notably, our analysis indicates that bisexual women are particularly vulnerable, reporting the highest prevalence of the four forms of sexual violence compared with nearly all other groups.

### Sexual violence and gender inequities

Our analysis revealed striking gender disparities in exposure to four types of sexual violence, disproportionately affecting young women, similar to previous studies [20, 22, 23, 27], and nonbinary + youth, as indicated by grey literature [25, 38], in comparison to young men.

The prevalence rates of being penetrated without consent, were particularly elevated among young women, as reported in other studies conducted in Sweden [23, 27]. Yet, the other reports indicated higher prevalence rates among men compared to our study, possibly reflecting methodological differences. The previous reports used broader measures like 'sex against one’s will,” while this study focused on more narrowly defined forms of ‘being penetrated without consent. Additionally, the results show that a considerable number of young women and nonbinary + individuals have been exposed to sexual violence on digital platforms, which has been reported in a previous study among youth in upper secondary school [23].

Furthermore, our study showed that a significantly greater number of young women and nonbinary + youth than young men had been physically assaulted against their will during sex. To our knowledge, this is the first time that this specific type of sexual violence has been examined at a population-based level. Thus, it is impossible to determine whether this violence has increased or decreased among young people. Nonetheless, the high levels and gender disparities are concerning.

### Sexual violence perpetrators

Young people reported that the main categories of sexual violence perpetrators included partners, acquaintances, and strangers, which aligns with previous research [39]. Our study found few statistically significant differences between young men and women regarding the reported categories of sexual violence perpetrators. This suggests that although there is a gender difference in exposure to sexual violence, the distinction between genders is less pronounced regarding the categories of perpetrators.

### Inequities across sexual orientations

The findings of our study revealed significant disparities in the four types of lifetime exposure to sexual violence across sexual orientations. Compared with young heterosexual men, young bisexual, lesbian and heterosexual women, as well as young gay and bisexual men, had greater odds of experiencing three out of four types of sexual violence. Previous international studies have highlighted the elevated risk of sexual violence against LGB people [30, 31], particularly bisexual individuals [40, 41]. We identified young bisexual women as a particularly vulnerable group for experiencing sexual violence, which concurs with previous research [29, 42]. While the reasons for the increased vulnerability among bisexual women have not been fully established, research indicates that they frequently encounter biphobia through negative comments, stereotypes and prejudices related to their sexuality [43]. Studies have also found a connection between bisexual stigma and a heightened risk of sexual violence and abuse [10, 43].

### Strengths and limitations

A significant strength of this study is its use of a stratified, population-based, cross-sectional survey with random selection supported by rigorous validation to ensure robustness. By disaggregating data on gender identity and sexual orientation, this study offers new insights into the victimisation of four types of sexual violence in Sweden. However, because it is a cross-sectional study, causality cannot be established. The response rate for our study was 23.7%, which limits the generalisability of the findings. Estimates for smaller subgroups in the study should be interpreted cautiously, as they may complicate the statistical precision of the analyses. In addition to the four types of sexual violence discussed in this study, many other forms of sexual violence can occur, such as verbal sexual harassment or situations where individuals are forced to penetrate others. Consequently, the overall prevalence of sexual violence among young people in Sweden is likely higher than what this study reported. The absence of a separate measure for “forced-to-penetrate” constitutes an important limitation and may contribute to the underestimation of certain forms of sexual violence, particularly among men. Therefore, the prevalence rates reported in this study should be understood within the context of these four specific types of violence. Furthermore, the study lacked data on the sex of the perpetrators; this information would have provided valuable insights, especially for bisexual women, who had 49.4 times greater odds of being penetrated without consent than heterosexual men did. While this high adjusted odds ratio (AOR) may partly reflect the small reference group of heterosexual men (n = 39), the stark difference in prevalence rates (38.7% vs. 1.2%) suggests a strong association.

### Implications for policy and future research

Despite the well-researched link between sexual violence and gender inequality, many preventative measures in Sweden lack a gender perspective [44]. As a gender-transformative approach is essential for challenging harmful masculinity norms [45], its inclusion must be prioritised. Policies and preventative measures must also adopt an intersectional lens, addressing multiple systems of oppression beyond gender inequality [11] – as a narrow or binary understanding of gender risks excluding marginalised groups such as nonbinary youth [46]. Equity requires explicit recognition of LGBTQI + individuals’ diverse vulnerabilities, with tailored measures for bisexual women, given their heightened risk.

It is essential to equip young people from an early age with the knowledge, resources, and practical skills necessary to build respectful relationships grounded in consent. Strengthening social institutions and enhancing professionals’ capacity, particularly within schools, is crucial. Sex education must be equitable and comprehensive, and provide structured opportunities to practice sexual communication. Another important aspect involves adults giving young people more opportunities for conversations and support concerning sex and relationships.

Furthermore, scientifically assessed programmes are crucial for ensuring effectiveness and scalability; however, they remain scarce, as do prevention efforts within university environments [44]. This underscores the need for more evaluations and targeted interventions across various fields. Moreover, prevention and support measures must also be extended to perpetrators, especially those who are neither prosecuted nor offered treatment [47]. Additionally, to address digital sexual violence, policies must ensure that preventive measures keep pace with technological advancements.

Advancing knowledge on sexual violence requires disaggregated data by gender and sexual orientation to identify disparities and inform inclusive policies. However, small subgroup sizes pose challenges. School-based studies may reach some youth but exclude others outside the education system. Innovative data analysis and qualitative research on LGBT and nonbinary youth are necessary to bridge these gaps. Furthermore, sexual violence jeopardises the possibility of safe, positive, and pleasurable sexual experiences [6], underscoring the need for research on its impact on young people’s sexual well-being.

Finally, the government must uphold the sexual and reproductive rights of young people, safeguarding them from violence. Increased funding for prevention and support is essential, as is a commitment to Agenda 2030 and national policies.

## Conclusion

In summary, our study reveals inequities in lifetime exposure to four types of sexual violence across gender identities and sexual orientations, revealing insights into the vulnerability of bisexual, lesbian, and heterosexual women, gay and bisexual men, as well as nonbinary individuals. To eliminate sexual violence in Sweden, measures involving stakeholders at all levels are needed, focusing on tailored prevention efforts and inclusive policies that address the vulnerabilities of these groups while promoting equity for all young people.

## Electronic supplementary material

Below is the link to the electronic supplementary material.

Supplementary Material 1

## Data Availability

The data supporting this study’s findings are available from the Public Health Agency of Sweden, but restrictions apply. The data was used under license for the current study and are not publicly available. However, data are available from the authors upon reasonable request and with permission of the Public Health Agency of Sweden.

## Abbreviations

IPV: Intimate partner violence
LGB: Lesbian, Gay, Bisexual
LGBTQI: Lesbian, Gay, Bisexual, Transgender, Queer, Intersex
PHAS: Public Health Agency of Sweden
SCB: Statistics Sweden
SDGs: Sustainable development goals
SRHR: Sexual and reproductive health and rights
WHO: World Health Organization
