# Correction: Sexual violence across gender identities and sexual orientations: a stratified, population-based, cross-sectional study among young people aged 16–29 years in Sweden

**DOI:** 10.1186/s12889-026-29650-w

**Published:** 2026-10-29

**Authors:** Matilda Löfström-Bredell, Eva Åkerman, Renita Sörensdotter, Sara Ström, Henrik Källberg, Marie Klingberg-Allvin

**Affiliations:** 1 Department of Women’s and Children’s Health, Karolinska Institutet, Stockholm, Sweden; 2 Centre for Gender Research, National Centre for Knowledge on Men’s Violence against Women, Uppsala University, Uppsala, Sweden; 3 Health Agency of Sweden (Folkhälsomyndigheten), Solna, Sweden

**Correction: BMC Public Health 25**,** 1878 (2025)**

**https://doi.org/10.1186/s12889-025-22970-3**

Following publication of the original article [1], terminology used in the article was updated. The term “Forced penetration” has been replaced with “Being penetrated without consent”, and “Nonconsensual sharing of sexual content” has been replaced with “Image-based sexual abuse”. These changes were made to align the terminology with current, more widely accepted usage in the field. In addition, the Declaration of Interest and Funding sections have been revised to provide clearer information on author affiliations and the funding agency’s role.

The original article [1] has been corrected.
