# Supplementary material for: Sexual violence across gender identities and sexual orientations: a stratified, population-based, cross-sectional study among young people aged 16–29 years in Sweden
Source: BMC Public Health. 2025 May 22;25:1878. doi: 10.1186/s12889-025-22970-3 (PMC12096477; doi:10.1186/s12889-025-22970-3)
Supplement: Supplementary file 1 — Supplementary Material 1 [file 12889_2025_22970_MOESM1_ESM.docx]

**Appendix 1. Survey items and response options**

| **Survey items** | **Response alternatives** |
| --- | --- |
| **Sexual violence and follow-up questions** | |
| Has anyone penetrated you with a penis, object, or fingers against your will (vaginally, anally, or orally)? | No |
|  | Yes |
|  | Not sure ^a^ |
| *Follow-up question: Who penetrated you against your will?* | *A current or former partner* |
|  | *A family member or relative* |
|  | *Someone close (other than above)* |
|  | *An acquaintance* |
|  | *Someone unknown* |
| Has anyone subjected you to physical violence against your will during sex? For example, slapped you, hit you, or put a chokehold on you. | No |
|  | Yes |
|  | Not sure ^a^ |
| *Follow-up question: Who subjected you to physical violence during sex?* | *A current or former partner* |
|  | *A temporary sex partner* |
|  | *An acquaintance* |
| Has anyone coerced you into engaging in any sexual acts via webcam/phone against your will? | No |
|  | Yes |
|  | Not sure ^a^ |
| *Follow-up question: Who coerced you into engaging in sexual acts via webcam/phone against your will?* | *A current or former partner* |
|  | *A family member or relative* |
|  | *Someone close (other than above)* |
|  | *An acquaintance* |
|  | *Someone unknown* |
| Has anyone spread sexual images or videos of you against your will or threatened to do so? | No |
|  | Yes |
|  | Not sure ^a^ |
| *Who spread, or threatened to spread, sexual images or videos of you?* | *A current or former partner* |
|  | *A family member or relative* |
|  | *Someone close (other than above)* |
|  | *An acquaintance* |
|  | *Someone unknown* |
| **Sociodemographic variables/ exposure variables** | |
| What is your gender identity? | Girl |
|  | Boy |
|  | Non-binary ^b^ |
|  | I don’t want to categorise myself ^b^ |
|  | I don’t know ^c^ |
| What is your sexual orientation? | Heterosexual |
|  | Homosexual |
|  | Bisexual |
|  | Pansexual ^c^ |
|  | Asexual ^c^ |
|  | I don’t want to categorise myself ^c^ |
| Gender identity & sexual orientation ^d^ | I don’t know ^c^  Heterosexual women  Heterosexual men  Bisexual women  Bisexual men  Lesbian women  Gay men |
| What is your relationship status? ^e^ | I am single |
|  | I am in a relationship |
|  | I am in several relationships |
|  | Other alternative |
| Have you had sex with someone else? | Yes |
|  | No |
| In the past 6 months, have you experienced discrimination and/or unequal treatment because of your sexual orientation? | No |
|  | Yes |
|  | Not sure ^f^ |

Note. ^a^ The response alternative ‘Not sure’ was included in the analyses presented in Tables 1 and 2 and excluded from all analyses presented in Tables 3–5, where it was dichotomised into ‘Yes’ /’No’.

^b^ Response alternatives were excluded from analyses presented in Table 3.

^c^ Response alternatives were excluded from analyses presented in Tables 3 and 5.

^d^ We developed a variable combining gender identity and sexual orientation with six categories.

^e^ Response options were dichotomised into “Single” and “In a relationship”, the latter also including “In several relationships”. “Other status” was excluded from all analyses.

^f^ Response option excluded from all analyses.

**Appendix 2. Statistics Sweden’s register variables, categories and descriptive statistics**

| **Registry variables provided by Statistics Sweden** | **Categories delivered from Statistics Sweden** | **Categories developed and utilized in analyses** |
| --- | --- | --- |
| Years of birth (Age group) | Birth years 1994 – 2007 | - 16 –19 years of age - 20–24 years of age - 25–29 years of age |
| Foreign background (Origin) | - Swedish-born with Swedish-born parents - Swedish-born with one foreign-born parent - Swedish-born with two foreign-born parents - Foreign-born | - Swedish-born with Swedish-born parents - Swedish-born with one foreign-born parent - Swedish-born with two foreign-born parents - Foreign-born |
| Household disposable income (2022) | Observations= 9,430  Mean: 4674.6 tkr^*^  Sd= 2709.1  Min= 1  Max= 9311  ^*^Tkr= One thousand Swedish Kronor | Five quintiles:   - Lowest - Low - Middle - High - Highest |
